# Conservation of Endangered *Lupinus mariae-josephae* in Its Natural Habitat by Inoculation with Selected, Native *Bradyrhizobium* Strains

**DOI:** 10.1371/journal.pone.0102205

**Published:** 2014-07-14

**Authors:** Albert Navarro, Simón Fos, Emilio Laguna, David Durán, Luis Rey, Laura Rubio-Sanz, Juan Imperial, Tomás Ruiz-Argüeso

**Affiliations:** 1 VAERSA, Centro para la Investigación y Experimentación Forestal (CIEF), Servicio de Vida Silvestre, Conselleria de Infraestructuras, Territorio y Medio Ambiente, Generalitat Valenciana, Quart de Poblet, València, Spain; 2 Centro de Biotecnología y Genómica de Plantas (CBGP) and Departamento de Biotecnología (ETSI Agrónomos), Campus de Montegancedo, Universidad Politécnica de Madrid, Pozuelo de Alarcón (Madrid), Spain; 3 Escuela de Ingenieros Técnicos Agrícolas, Universidad Politécnica de Madrid, Madrid, Spain; 4 CSIC, Madrid, Spain; Graz University of Technology (TU Graz), Austria

## Abstract

*Lupinus mariae-josephae* is a recently discovered endemism that is only found in alkaline-limed soils, a unique habitat for lupines, from a small area in Valencia region (Spain). In these soils, *L. mariae-josephae* grows in just a few defined patches, and previous conservation efforts directed towards controlled plant reproduction have been unsuccessful. We have previously shown that *L. mariae-josephae* plants establish a specific root nodule symbiosis with bradyrhizobia present in those soils, and we reasoned that the paucity of these bacteria in soils might contribute to the lack of success in reproducing plants for conservation purposes. Greenhouse experiments using *L. mariae-josephae* trap-plants showed the absence or near absence of *L. mariae-josephae*-nodulating bacteria in “terra rossa” soils of Valencia outside of *L. mariae-josephae* plant patches, and in other “terra rossa” or alkaline red soils of the Iberian Peninsula and Balearic Islands outside of the Valencia *L. mariae-josephae* endemism region. Among the bradyrhizobia able to establish an efficient symbiosis with *L. mariae-josephae* plants, two strains, LmjC and LmjM3 were selected as inoculum for seed coating. Two planting experiments were carried out in consecutive years under natural conditions in areas with edapho-climatic characteristics identical to those sustaining natural *L. mariae-josephae* populations, and successful reproduction of the plant was achieved. Interestingly, the successful reproductive cycle was absolutely dependent on seedling inoculation with effective bradyrhizobia, and optimal performance was observed in plants inoculated with LmjC, a strain that had previously shown the most efficient behavior under controlled conditions. Our results define conditions for *L. mariae-josephae* conservation and for extension to alkaline-limed soil habitats, where no other known lupine can thrive.

## Introduction

Lupines are an isolated lineage of legumes within the Genistae tribe. The genus *Lupinus* comprises *ca.* 280 species of annual herbs and herbaceous and woody perennials shrubs, some of which are cultivated as green manure and pulse crops. The majority of *Lupinus* species are distributed in the New World and only fifteen species are native to the Old World, in areas surrounding the Mediterranean [1]–[4].

The capacity of lupines to establish a symbiosis with rhizobia and effectively fix nitrogen in root nodules allows these plants to thrive in nutrient-poor soils. Lupines are nodulated by slow-growing bacteria classified within the *Bradyrhizobium* genus, and most of these strains broadly cluster within the *B. japonicum* lineage. Genetic studies have revealed that European *Lupinus* spp. are nodulated by the two dominant species within the genus *Bradyrhizobium*, *B. canariense* and *B. japonicum* [5]–[7].

Based on botanical characteristics, a new lupine species, designated *Lupinus mariae-josephae* (Lmj), singularly thriving in alkaline soils with high pH and Ca^2+^ content, has been identified in the province of Valencia, in Eastern Spain [8]–[10]. More recent phylogenetic studies have confirmed the unique taxonomic position of Lmj and established it as a distinct evolutionary line among the Old World lineages, although its exact taxonomic position is still poorly resolved [11].

The *L. mariae-josephae* endemism, contrary to all hitherto described Old- and New-World lupines, which are preferentially adapted to acid soils [12]–[15], thrives on alkaline soils of the “terra rossa” type, meaning red or reddish-brown soils on carbonate rock [9]. Despite continued efforts on the part of researchers and technicians from the Centro de Investigación y Experimentación Forestal (Quart de Poblet, Valencia), just a few populations of the plant have been located in four geographic regions of Valencia province [9], [10]. Most of these have been given a “microreserve” status for conservation of the species [16]. The Lmj endemism is present within an area of about 700 km^2^ (70,000 Ha) at an altitude of 200–400 m above sea level in the Valencia province. However, within this area, Lmj plants only grow in a very limited number of reduced-surface patches that contain small populations in the hundreds and up to several thousand plants in years with optimal climate conditions [9], [10].

Only recently have studies on the rhizobia nodulating Lmj been undertaken. Sanchez-Cañizares *et al.* [17] characterized Lmj-nodulating bacteria from soils of a specific site in the Llombai (Valencia) area, and concluded that they belong to a new *Bradyrhizobiun* lineage unrelated to *B. canariense* or *B. japonicum*. Later on, a wider study on the genetic diversity of Lmj-nodulating bacteria characterized up to nineteen groups of *Bradyrhizobium* strains belonging to up to six genospecies that were able to nodulate Lmj plant populations in the Valencia province [18].

The reduced *L. mariae-josephae* habitat and the plant's characteristic soil chemical requirements place this lupine species in danger of extinction and emphasize the need for research directed towards its conservation. The goal of this work was to take advantage of our knowledge of the Lmj symbiosis and of our collection of Lmj endosymbiotic bacteria, in order to explore the possibility of extending the growth area of Lmj plants to additional patches in the “terra rossa” soils of Valencia, and to other apparently similar soils in the Iberian Peninsula and Balearic Islands, thereby contributing to ongoing efforts directed to conservation of the species. As part of the present work, experiments leading to selection of efficient nitrogen fixing strains for inoculant production and *in situ* field trials with inoculated seeds were performed.

## Materials and Methods

### *L. mariae-josephae* endosymbiotic bacteria isolation and screening in soils

All field work on the endangered species *Lupinus mariae-josephae*, both in micro-reserves and in unprotected, communal land was carried out under permit and supervision from the relevant regional authority (Generalitat Valenciana, Conselleria de Infraestructuras, Territorio y Medio Ambiente, Servicio de Vida Silvestre, VAERSA, Centro para la Investigación y Experimentación Forestal (CIEF) and VAERSA, Avda. Comarques del Pais Valencià, 114. 46930 Quart de Poblet, Valencia), whose conservation experts (AN, SF and EL) actively collaborated in this work and are co-authors of this communication. Soil samples from areas outside the Valencia region were taken from unprotected, communal land, for which no permit was necessary. The GPS coordinates for all *L. mariae-josephae* populations and soil sampling sites are listed in Table 1.

**Table 1:** Presence of *Lupinus mariae-josephae* nodulating bacteria in “terra rossa” and red alkaline soils in Spain†.

Origin of Soils (coordinates)	Plant Appearance	Nodulation				Soil Chemical Characteristics				Soil Type
		nodule number▵	Nodule color	pH	Ca*	Mg*	N#	P*	K*	
Llombai plant patch	green	3.6±0.5	red	7.98	7,720	120	3.8	7.7	310	terra rossa
(39.3189373°, −000.5639983°)										
Xàtiva plant patch	green	3.5±0.4	red	8.17	6,880	110	2.2	<5	170	terra rossa
(38.9488459°, −000.5305723°)										
Outside Llombai plant patch	yellow/green	1.3±0.4	reddish	7.65	6,120	110	3.0	<5	320	terra rossa
(39.3189373°, −000.5639983°)▾										
Outside Xàtiva plant patch	yellow	0	*-*	7.47	2,960	90	1.2	<5	190	terra rossa
(38.9488459°, −000.5305723°)▾										
Field trial site	yellow/green	1.5±0.5	reddish	7.98	6,840	200	3.2	5.9	680	terra rossa
(39.2918111°, −000.6591528°)										
Mallorca 4	yellow	0	-	8.36	6,990	480	1.1	<5	1150	terra rossa
(39.3582222°, 002.8224722°)										
Mallorca 6	yellow	0	-	9.09	5,600	1180	1.4	<5	1690	terra rossa
(39.3635833°, 002.8902222°)										
Pozuelo del Rey	yellow	0	-	7.9	5,550	60	0.1	<5	190	terra rossa
(40.3728306°, −003.3259472°)										
Aldeanueva de la Serrezuela	yellow	0	-	7.49	2,750	110	0.1	<5	150	terra rossa
(41.4569722°, −003.7812083°)										
Fresno de la Fuente	yellow	0	-	7.86	6,930	200	0.1	<5	790	terra rossa
(41.3937444°, −003.6442917°)										
Alcala de Guadaira	yellow	0	-	7.95	5,030	130	0.1	8.3	490	terra rossa
(37.3270278°, −005.7963333°)										
La Luisiana	yellow	0	-	8.09	7,650	260	0.1	27	1240	red
(37.5248333°, −005.4024167°)										
Calzadilla de los Barros	yellow	0	-	7.87	7,260	200	0.1	<5	460	red
(38.3488556°, −006.3217944°)										

† Symbiotic bacteria were screened using Lmj trap-plants in sterile Leonard jar units employing 50 g of soil per jar and 8 jars (16 plants) per soil (see Materials and Methods).

▵ Average of nodules per Leonard jar ± standard error.

* Expressed in mg.kg^−1^.

# Expressed in g. kg^−1^.

▾ 200 meters outside the plant patch.

**Figure 1:**
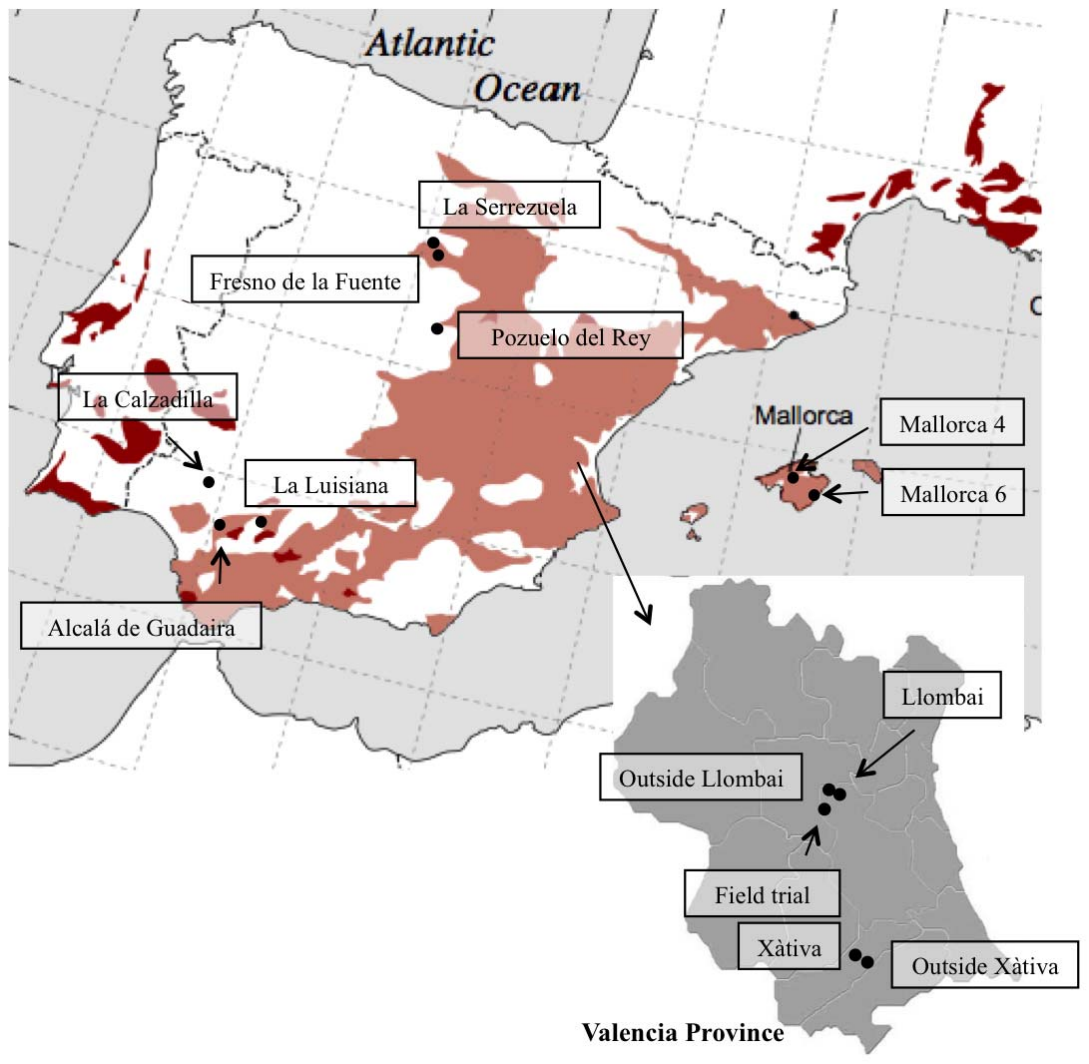
Map of Spain with sampling sites indicated by black stars and their named location (see Table 1). The distribution of Red Mediterraean and reddish brown soils is indicated by colors (after Muhs et al. [22]).

The presence of *L. mariae-josephae* endosymbiotic bacteria was investigated in “terra rossa” or alkaline red soils from Iberian Peninsula and Balearic Island (Fig. 1, Table 1) using Lmj as trap plant, as previously described [17], [18]. The limiting number of available Lmj seeds precluded precise estimations of Lmj-nodulating bacteria by the Most Probable Number method [19], and their presence in soil samples was qualitatively evaluated by determining the number of nodules produced in 18 plants grown from germinated sterilized seeds in Leonard jar units (15×30 cm). Leonard jars contained 50 g soil each in sterile vermiculite, N-free Jensen's solution in the lower chamber, and two plants were planted in each. Plants were examined for nodulation and plant and nodule colour after *ca.* 6 weeks. Soil chemical composition was determined by standard procedures [20].

### Field trials

Two nitrogen-fixing, symbiotically-efficient bacterial strains, LmjC and LmjM3, previously isolated from “terra rossa” soil from the known plant patches in Valencia region, were selected as inoculant strains [17], [18]. Peat-based inoculants were prepared for field trials using these strains and following standard methods for peat-based inoculant preparation [19]. A black, neutral peat from Padul (Granada, Spain) was used, as its carrier properties have been shown to be optimal for rhizobial survival [21]. Bacterial cultures (10^8^ cfu.ml^−1^) were thoroughly imbibed in finely ground, sterilized peat (5 ml culture.10 g^−1^ peat) shortly before their use for inoculation in order to maintain inoculum viability. For field assays, Lmj seeds were scarified by overnight soaking in just-boiled water and germinated on agar plates. Germinated seeds were inoculated with the peat-based inoculants obtained as described above and following standard techniques [19]. Control plants were inoculated with a sterile peat preparation.

The field trial site was located in Lloma de Coca, a municipality distant *ca.* 10 km from the Llombai Lmj plant patch [17], [18], indistinguishable in its geo-botanic and edaphic characteristics, but where no Lmj plants have ever been observed [9], [10]. This site is located in unprotected communal land for which no special permit was necessary, but under the direct supervision, and with the collaboration, of conservation experts from the competent conservation authority who are co-authors of this work (AN, SF and EL). The GPS coordinates of the field site are listed in Table 1.

**Figure 2:**
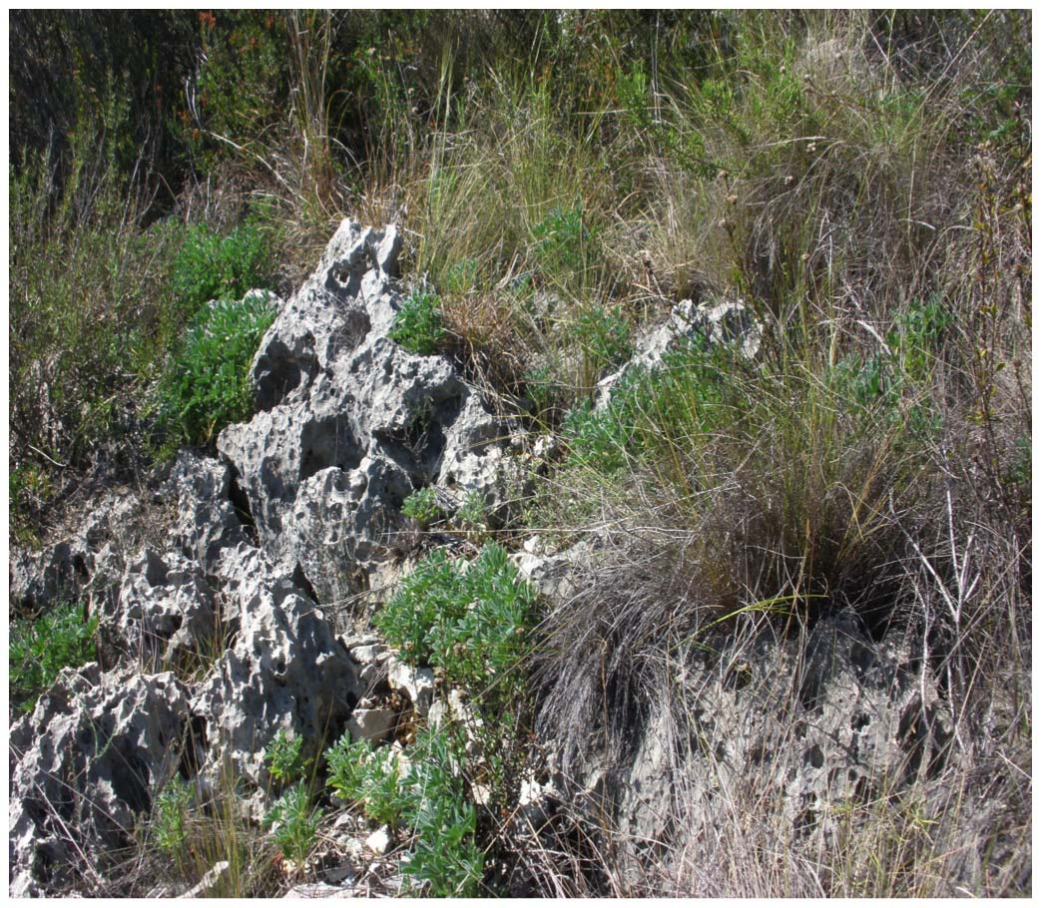
*L. mariae-josephae* plants growing in the cracks of weathered limestone, in its “terra rossa” natural habitat (Llombai, Valencia).

**Figure 3:**
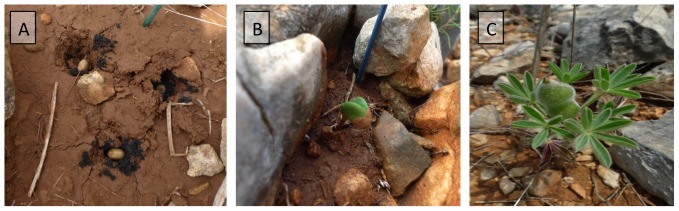
Successive steps in field trial. A: spot with 3 germinated seedlings; B: sprout in a planted spot; C ripe plant with sheaths containing seeds.

The adverse edaphic conditions of the area, where very small soil spots (200–2,000 cm^2^) appear within cracks of the limestone (Fig. 2), bar setting-up any properly designed experimental distribution. Instead, experiments were performed by carefully planting groups of 3 germinated seeds in soil spots outlining three tiny holes, each containing the peat inoculant. About 30 spots were chosen for each of the three treatments (inoculation with two different strains plus an uninoculated control), making sure that nascent roots were adequately covered by soil (Fig. 3). Experiments were started in early October in all cases and ended in early Spring, soon after pod filling but before pods dried and opened.

Inoculant strains were recovered from rhizospheric soil at the experimental sites and were identified after determining their *recA* sequences as previously described [17], [18].

### Statistical analyses

One-way and two-way ANOVA analyses were carried out with Kaleidagraph 4.5 (Synergy Software, Reading, PA). Fisher's Least Significant Difference (LSD) post-hoc test at the α = 0.05 level was used to evaluate how plant variables in Tables 2 and 3 responded treatments (bacterial strains used as inoculum).

**Table 2:** Evaluation of symbiotic efficiency of *Lupinus mariae-josephae* bradyrhizobia strains.

Strain	Nodulation▵ (nodules/plant)	BNF▾	Dry weight (gr/plant)
LmjC	20.8±1.5 a*	15.22±1.85 a	7.85±0.33 a
LmjH2p	15.8±1.5 ab	9.46±1.47 b	5.05±1.01 ab
LmjM1	16.5±0.6 ab	11.25±2.62 ab	5.83±0.78 ab
LmjM3	19.8±1.1 a	14.80±2.95 a	6.40±0.22 ab
LmjG2	11.3±1.0 b	5.82±1.35 b	4.04±0.67 b
LmjG3	12.3±1.3 b	7.30±0.66 b	4.74±0.58 b
LmjX7	9.5±1.4 b	3.06±0.84 b	3.44±0.67 b
LmjX10	11.9±1.1 b	3.71±0.84 b	3.67±0.97 b

▵ Plants were grown in Leonard jar units and were inoculated with bacterial culture at similar concentration (10 ml of 10^7^–10^8^ cells/ml per unit).

▾ BNF: Biological Nitrogen Fixation, expressed as µmoles acetylene per hour and mg nodules ± standard error.

* Means (± standard error) followed by different letters were significantly different (α = 0.05) when compared by Fisher's LSD post-hoc test.

**Table 3:** Effect of inoculation on *L. mariae-josephae* seedling performance in field trials performed in two succesive seasons.

Inoculant	Seedlings sown	% survival	Pods▾	Average seeds per plant	Total seeds
Season 2011–2012					
LmjC	96	53 a*	4.37±0.43 a	12.72±1.24 a	649
LmjM3	84	58 a	1.87±0.09 b	4.65±0.38 b	228
Control	78	28 b	1.27±0.09 c	2.59±0.25 c	57
Season 2012–2013					
LmjC	99	39 a	9.38±0.85 a	26.20±2.7 a	1,022
LmjM3	99	33 a	2.54±0.19 ab	5.39±0.51 ab	178
Control	99	16 b	1.56±0.22 b	3.12±0.83 b	50
Season comparison					
*F*		17.69	6.44	4.37	
*P*		0.05	0.01	0.03	

▾ Mean per plant ± standard error (σ/√n).

* Column values were analyzed independently for each season. Within a given season, those values followed by different letters were significantly different (α = 0.05) when compared by Fisher's LSD post-hoc test.

## Results

### Evaluation of the presence of Lmj-nodulating bacteria in soils

The existence of Lmj-nodulating bacteria was examined in “terra rossa” and in “red soils” from the Valencia Lmj region and from other locations in the Iberian Peninsula or Balearic Islands (Table 1, Fig. 1). “Terra rossa” soil sites were chosen within areas well known to contain “terra rossa” soils found along many of the continental shores and islands of the Mediterranean basin [22]. The chemical composition of these soils is quite diverse but all have pH values near 8 and elevated Ca^2+^ concentrations (Table 1) as had also been previously reported for the Lmj area in Valencia [17], [18].

The presence of Lmj-nodulating bacteria in soil samples was determined by using Lmj as trap-plant. Analysis of nodule numbers revealed that Lmj-nodulating bacteria were present in Valencia “terra rossa” soils from Lmj plant paches in the Llombai and Xàtiva locations (Table 1), at levels similar to those previously determined [17], [18]. Outside the Llombai Lmj plant patch (*ca.* 200 m away), Lmj-nodulating bacteria were also found but, within the limitations of the semi-quantitative method employed, resulting in much lower nodule numbers (Table 1). Similar low levels of Lmj-nodulating bacteria were detected in soil samples from the assay field. In all cases, nodules were reddish and appeared not to fix enough nitrogen to yield plants as green as those grown in soils from Lmj-plant patches, suggesting that numbers of nodulating bacteria may be limiting. Endosymbiotic bacteria were isolated from those nodules, their *recA* gene sequences were determined and phylogenetic trees were derived from those, all as previously described [17], [18]. As expected, these analysis identified them as *Bradyrhizobium* spp. (*L. mariae-josephae*) [17], [18]. Regarding the Xàtiva Lmj plant patch, however, no Lmj-nodulating bacteria were found in a “terra rossa” soil spot distant *ca.* 200 m from the Lmj population. All together, these results suggest that low numbers of Lmj-nodulating bacteria are found in Valencia “terra rossa” soils outside the Lmj plant patches.

Similar greenhouse experiments using Lmj trap-plants were performed with eight “terra rossa” or red soils from different geographical sites in Spain (Fig. 1). No nodules were detected in plants grown in the six “terra rossa” soils tested, which include four from the Iberian Peninsula and two from Mallorca (Balearic Islands), nor in the two alkaline red soils examined. These results suggest that Lmj-nodulating bacteria are absent (or in undetectable numbers) in “terra rossa” or alkaline red soils outside the Valencia *L. mariae-josephae* endemism region.

### Selection of efficient Lmj strains for inoculant production

Greenhouse tests were carried out with eight previously selected, Lmj-symbiotic strains representative of isolates from the four known Lmj plant patches [18]. Number of nodules, biological nitrogen fixation, and whole-plant dry weight were determined 6 weeks after inoculation, and results are shown in Table 2. *L. mariae-josephae* strains LmjC and LmjM3 showed the highest efficiencies, as indicated by all three measured variables, and were thus chosen as inoculants for field trials.

Previous to their use in field trials, peat-based LmjC and LmjM3 inoculants were produced and tested under standard agricultural conditions in regular soils from the CIEF Experimental Station located in Valencia coast. This is a typical acidic-to-neutral agricultural soil area, where Lmj plant reproduction trials had previously been unsuccessful (data not shown). After inoculation with the selected Lmj strains, plants developed but did not reach maturity, and eventually wilted. Nevertheless, inspection of their root systems revealed the presence of few (5 to 10 per plant) reddish root nodules in the inoculated plants. No nodules were observed in uninoculated control plants.

### Field trials

The field trial site is located in an area indistinguishable in its geo-botanic and edaphic characteristics from the Llombai Lmj plant patch area (Table 1), but where no Lmj plants have previously been observed. Adverse edaphic conditions in the whole area made it impossible to set-up any properly designed experimental distribution. Instead, experiments were performed as described in detail in Material and Methods section. Trials were carried out in three successive seasons (2010–2013).

In the 2010/11 field trial, unseasonal frost decimated test plants and no nodules could be recovered from deep, hard-to-reach root systems. However, the inoculant strains could be recovered from soils after the experiment, suggesting that both LmjC and LmjM3 had survived in “terra rossa” soils in the Lmj distribution area.

Results from the 2011/12 and 2012/13 trials are shown in Table 3. In both trials, where *ca.* 90 pre-germinated seeds were planted, much higher plant survival (about double) and, specially, higher reproductive success (either pods or grain production) were achieved with the rhizobial inoculation. However, there were significant differences between seasons for all variables studied (survival, pods per plant and seeds per plant) with *F* (*P*) values of 17.69 (0.05), 6.44 (0.01), and 4.37 (0.03), respectively (Table 3). Therefore, the effect of inoculant strains was analyzed independently for each of the seasons. Clear differences in the inoculation response occurred in both seasons, especially with strain LmjC, with a higher response in 2012/13 for this strain. Here, inoculation resulted in over 20 times more seeds than the uninoculated control. Some of the observations deserve a specific comment. First, strain LmjC plainly outperformed LmjM3 strain in both trials. In fact, for the 2012/13 season, the effect of LmjM3 inoculation on pod and seed yield was barely significant (Table 3), although inoculation clearly had a positive effect on survival, and thus in total seeds collected. A second observation is that, for the high-performing strain LmjC, survival and reproduction success were not correlated, since in the 2011/12 season viability was higher than in 2012/13, but seed production was lower. Both inoculants increased survival rates in a similar way, but plant vigor and biomass were much higher for LmjC-inoculated plants (data not shown). This resulted in grain production that was higher by three- to six-fold in plants inoculated with LmjC strain, suggesting that LmjC is probably the inoculant of choice for further field trials. Finally, it also can be pointed out that results shown in Table 3 suggest that inoculation increases plant conservation. As it is particularly evident in the 2012/13 season, grain production by plants inoculated with LmjC strain multiplied ten-fold the number of seeds planted (1,022 from 99), a high enough number to ensure population viability, clearly not the case with non-inoculated plants (50 from 99). A similar pattern (649 seeds from 96; and 57 seeds from 78, respectively) was observed in the 2011/12 season. These results support the idea that seedling inoculation can be an adequate strategy to increase conservation of an endangered legume.

## Discussion

*Lupinus mariae-josephae* (Lmj) thrives in a habitat with unique characteristics and constitutes an endemism in some soils of the “terra rossa” type in Eastern Spain. Within the Valencia province, Lmj has only been identified in four reduced plant patches. The present study shows that a successful reproductive cycle of this lupine species outside the known plant patches was absolutely dependent on seedling inoculation with effective bradyrhizobia. The term “terra rossa” became a common denomination for all hard limestone-derived red soils in regions with a Mediterranean climate [23]. They are a type of red clay soils produced by either the weathering of limestone rock or by total or partial replacement of limestone bedrock by dissolved aeolian dust [22], [24]. Upon weathering, environment chemical oxidation of Fe^2+^ occurs, with formation of Fe^3+^ hydroxides and oxides (rusting), that give clay its characteristic reddish-brown coloration [25].

Regardless of the origin of terra rossa soils from Eastern Spain, the ability to grow on alkaline-limed soil sets the Lmj lupine species apart from all other lupines, which preferentially or exclussively thrive in acidic soils [12], [26]. However, within its area of distribution only a reduced number of small Lmj populations have been found, which placed the species within the Valencia endangered species list ([9], [10], unpublished data). The climatic or edaphic factors within this region that restrict Lmj populations to the terra rossa soils of Eastern Spain, and there only to small populations, are not known. Therefore, strategies addressed to promote its conservation were needed. As a first approximation to protect the known populations, “microreserves” promoted by the European Union [16] have been established for some of them. Furthermore, given that legumes rely on their symbiosis with root-nodule bacteria, and since the presence of these symbiotic bacteria in the soil could potentially be a limiting factor [27], [28], this aspect was investigated in the present work.

In the Valencia area where natural Lmj populations are found, mature “terra rossa” soil is scarce and appears filling the cracks of weathered limestone (Fig. 2). This severely hampers recovery of root systems and root nodules. However, by using trap plants, we were previously able to recover Lmj-specific bradyrhizobia that can form nitrogen-fixing root nodules from soil samples from the known Lmj plant patches [17], [18]. In this study, using a similar methodology, the presence of Lmj-nodulating bacteria has also been shown in samples of soils from outside the plant patches, such as 200 m away from the Llombai plant patch and from Lloma de Coca, the location of the field trials, although forming remarkably lower numbers of nodules than within the plant patches, where they may have been enriched by the symbiosis (Table 1). The low numbers of nodules observed may be explained by a long absence of Lmj plants and by low rhizobial survival rates in the soil.

Although Lmj is nodulated by bradyrhizobia of very different genotypes [17], [18], which even belong to different *Bradyrhizobium* species (Duran et al 2014, data not shown), no Lmj-nodulating bacteria were detected in terra rossa soils or alkaline red soils from other geographical locations in the Iberian Peninsula or Balearic Islands. This is consistent with the absence of Lmj lupines in these soils, possibly because of edapho-climatic differences with regard to those in the Valencia endemic region, and therefore the absence of selection for Lmj-specific bradyrhizobia. Our previous phylogenetic analysis [17], [18] showed indeed that *L. mariae-josephae* growing in the “terra rossa” alkaline soils of Eastern Spain specifically select particular subpopulations of endosymbiotic bacteria, which are phenotypically and phylogenetically different from bacteria nodulating *Lupinus* spp. thriving in acid soils [5]–[7], [29]. In this respect, it is worth noting that traditional lupin rhizobia have long been known to be very sensitive to high pH [30], and that their numbers are very low in alkaline soils. This is consistent with the observed poor root growth and reduced nodulation of lupins at pH levels above 6.0 [12], [26], for all tested species except Lmj.

The availability of a collection of Lmj symbiotic strains [17], [18] allowed us to carry out symbiotic efficiency experiments in the greenhouse and select strains LmjC and LmjM3 as the most highly effective for *in situ* field inoculation experiments. The adverse edaphic conditions present in the Lmj growing area (Fig. 2), barred the set-up of any properly designed experimental distribution. Even with these shortcomings, a simple planting scheme, involving germinated seeds in appropriate spots (Fig. 3), showed, in two successive seasons (2011/12 and 2012/13) much higher survival and, especially with the LmjC strain, reproductive success upon rhizobial inoculation. Although both inoculants, LmjM3 and LmjC, increased survival rates in a similar way, plant vigor and biomass were significantly higher for LmjC-inoculated plants.

There were clear differences in yields between seasons, and these can be explained by climate variables and biological traits of the plant [31]. Even taking into account the interannual fluctuations of crop yields, the observed yield increase, ranging, from six to ten seeds per sown plant (with the LmjC inoculant), supports the hypothesis that the presence of appropriate bacterial symbionts in the soil is the main factor limiting expansion of the known, reduced Lmj plant patches. Therefore, the seedling inoculation strategy described in this work is appropriate for directed colonization of new areas with *L. mariae-josephae*. With appropriate handling, this, in turn, will ensure conservation of the species. To our knowledge, this represents the first example documenting the possibilities of inoculation technology for the survival and recovery of an endangered legume species in its natural environment. Future monitoring of the newly funded populations will determine the feasibility of increasing the Lmj distribution area to other “terra rossa” and, perhaps more importantly, other basic soils, from which lupines, widely recognized as pioneer plants that play important roles not only for seed and forage, but also for ecosystem recovery, have so far been excluded.

Results presented in this paper suggest that plant inoculation with specific, symbiotically-efficient rhizobia might be a worthwhile addition to conservation efforts for other endangered legumes. In most cases, this strategy would require, as for *L. mariae-josephae*, the previous isolation and characterization of these specific rhizobia, and we are currently testing this concept with other endemisms, such as *Astragalus nitidiflorus*, a severely endangered legume from Southeastern Spain, previously thought to have become extinct [32].
